# The Value of Chemistry in Dentistry

**Published:** 1905-04-15

**Authors:** George M. Heath


### THE VALUE OF CHEMISTRY IN DENTISTRY.

BY GEORGE M. HEATH.

Read before the Central Michigan Dental Society, March 28, 1905.

Frequently to-day those of you who have been practitioners of dentistry for many years feel disheartened as you find how difficult, almost impossible, it is to keep ahead of your rapidly-growing, advancing science.

The ones who are still enthusiastic and industrious, it is true, by attentive and careful reading, by contact with the younger or earnest workers, especially with those whose main field is laboratory research, can remain sympathetic and appreciative.

But the great majority have neither the knowledge, the skill, the training, nor generally the time to do the actual scientific work which makes them feel and know they are in this direction on a par with their pupils, their successors, the younger generation. For this reason, and yet reluctantly and with the deep sentiment that it should not be, they "take a back seat" in discussions and papers, and budding practitioners appear unduly to them, oftentimes, to fill their places.

Of course, to the younger generation (full of the new microscopy, chemistry, and bacteriology), there will come what they consider new lights and new discoveries. To these discoveries will possibly rest the future success of dentistry—not necessarily in a mechanical way, but along the line of medicine and chemistry.

Dentistry is a specialty of medicine, and more scientific knowledge concerning the action of drugs in diseases conditions of the oral cavity will be found essential. Every dentist should be capable of making a complete diagnosis of all oral and systemic diseases that are connected with oral symptoms and able to treat them just as intelligently, if not more so, than a physician.

But in order to diagnose a case accurately and prescribe intelligently, one must understand the chemical nature of the organs, their constituents, and the chemical action of the drugs and chemicals used in the treatment of the different diseases.

Not until a close connection was established between chemistry and medicine did any decided advance occur in the latter; and by its means, the medicine of the future will undoubtedly be still further enlightened and extended.

Physiological chemistry promises much even in the treatment of disease, for it is beginning to be seen from the investigation of the molecular constitution of different bodies that there exists a distinct connection between their specific atomic grouping and their physiological action. And even in some of the most interesting investigations of the pathology of the present day—those connected particularly with the causation of infectious diseases—physiological chemistry will, without a doubt, take a most prominent place; for there is every reason to believe that the micro-organisms which play so important a part in these diseases really act by virtue of a ferment working virus or zymosis of some kind which accompanies them or is generated by them. The hope therefore may be expressed that further investigation of their chemical properties may lead to a knowledge of how to stamp them out or to thwart their activity.

Dentistry offers a very wide field for the investigations of the chemist. The chemistry of cements, alloys, and amalgams in analytical chemistry, and the saliva, teeth, bacteria, etc., in physiological and bacteriological chemistry are each of sufficient importance to warrant more careful chemical investigation.

In the chemistry of saliva, a very common yet important factor that the dentist has to deal with, we find the diastatic ferment, ptyalin, mucin, and the chlorides of sodium and potassium, also traces of albumen, fat, potassium sulfocyanide, sulphates and phosphates of the alkalies and alkaline earths, and sometimes even in normal saliva, urea and ammonium nitrate.

The oral secretions, when normal, are slightly alkaline, but under some constitutional disturbances such as catarrh of the mouth and intestinal tract, carcinoma of the liver, diabetes, long fasting, the eating of substances difficult of digestion, and at times under the effect of constitutional remedies, the saliva will become acid in reaction, which in itself is destructive to tooth substance and will start caries by attacking the enamel and by chemically depositing upon the least exposed surface of the teeth, substances that favor the rapid development of bacteria and their injurious effect.

Saliva from different individuals may show a constant difference in alkalinity, although it varies only within narrow limits; and while showing within certain limits in the same individual a constant degree of alkalinity, there is a decided and constant difference in different individuals, but no constant corresponding difference in diastatic action. In diabetes, the saliva may be acid and contain lactic acid, but this is exceptional.

Leaving for the present the saliva and passing on to the composition of the bone, we find the latter to consist of an organic substance, ossein, a modification of collagen, intimately combined with a mineral substance, the so-called bone earth, in the proportion of about thirty of the ossein to seventy of the bone earth. This bone earth is a mixture of several salts, the chief of which is tribasic phosphate of lime; there is besides about nine percent calcic carbonate, three percent calcic fluoride, and one and seven tenths percent of magnesic phosphate.

The general composition of normal undried bone without any separation of fat, marrow, or blood lymph, may be given as:

| | | |
|---|---|---|
| Water | 50 | percent. |
| Fat | 15.75 | " |
| Ossein | 11.40 | " |
| Bone Earth | 21.85 | " |

The different bones of the skeleton differ slightly one from the other in chemical composition, and the organic constituents are said to increase with age, but the increase is only very slight, the great fragility being due to the increased porosity of the bone in old age.

The teeth with their three distinct tissues—dentine, enamel, and cement or crusta petrosa, are in great part epidermic structures.

The cement has the structure of bone, and in its composition it is almost analogous thereto, its organic and inorganic constituents having the relative proportions of thirty to seventy.

Dentine, like bone, contains an animal matrix impregnated with earthy salts. The animal matrix is of the nature of ossein, but the walls of the canaliculi appear to have special investments of a body resembling keratin or elasticin. Dentine is four percent poorer in water than bone, and contains twenty-six to twenty-eight percent organic substances. Reckoning the carbonate and phosphate of lime together, Hoppe Seyler gives the composition as:

| | |
|---|---|
| Ca 10 C O 3-6 (PO4) | 72.06 percent. |
| Mg HPO 4 | 0.75 " |
| Organic Substances | 27.70 " |

According to Bibra, the following is the composition of the dentine of men and women respectively:

| | Adult Man | Adult Woman |
|---|---|---|
| Organic Matter, Ossein and Vessels | 20.42 | 27.61 |
| Phosphate of Lime | 67.54 | 66.72 |
| Carbonate of Lime | 7.97 | 3.36 |
| Phosphate of Magnesia | 2.49 | 1.08 |
| Other Salts (Na Cl, etc.) | 1.00 | 0.83 |
| Fat | 0.40 | 0.58 |

In the enamel the ash averages ninety-five to nine-seven percent; in the young infant, it varies between seventy-seven and eighty-four percent. In the dog, there appears to be no organic material at all. Its average composition may be thus stated:

| | |
|---|---|
| Water and Organic Substances | 3.6 |
| Calcic Phosphate and Fluoride | 86.9 |
| Magnesic Phosphate | 1.5 |
| Calcic Carbonate | 8.0 |

ENAMEL AND DENTINE COMPARED.

| | Enamel | Dentine |
|---|---|---|
| Organic Substance and Water | 3.60 | 27.70 |
| Inorganic Substance | 96.40 | 72.30 |

IN 100 PARTS ASH:

| | Enamel | Dentine |
|---|---|---|
| Calcic Phosphate | 93.35 | 91.32 |
| Calcic Carbonate | 4.80 | 1.61 |
| Calcic Oxide | 0.86 | 5.27 |
| Magnesic Carbonate | 0.78 | 0.75 |
| Calcic Sulphate | 0.12 | 0.09 |
| Oxide of Iron | 0.09 | 0.10 |

All the important animals of the world have teeth, not by accident, nor for ornament, but for service in properly masticating food necessary to preserve life, and it is a fact that all animals living in and eating the food of their natural habitat have good teeth throughout their natural life.

You will notice that in comparing the percentage of organic substances in the enamel of dogs' teeth and animals' teeth in general, with that of a human being, the former contains practically no organic matter. That may be looked upon as the cause of the greater decay of the teeth of human beings than in animals. For it seems reasonable that this organic substance, stimulated by the action of the bacteria of varying sizes and forms, intermixed with particles of food which go to form a part of the tartar that deposits on the teeth, is destroyed gradually by this bacteria.

The decay is certainly not due to a difference in the composition or structure of the dentine. Alkalies have a tendency to destroy fermentation, whereas acids do not. It is true that acids attack the enamel and dissolve it, allowing the dentine to become inoculated with other substances and, owing to its large percentage of organic matter, it readily decays.

The continual use of the organs gives the bacteria very little chance to pass the incubative period without being ousted from their location before they can cause any acid formation to attack the enamel of the teeth. This is Nature's prophylaxis. The teeth of the enlightened will be found poorer and more prone to decay than those of the less civilized, whose diet usually requires some vigorous acts of mastication. The prophylactic condition of the teeth is far better on the side of the mouth most used, even the teeth of the tobacco-chewer will be found in much better condition on his "tobacco-side," excepting, of course, the usual wear.

It appears to me that as long as the health of all organs and parts of the body are dependent upon its general health we might look for diseases of the teeth to appear through metabolism, and in this case your best efforts will fail to cure them until you have cured the seat of the trouble.

When the ailment or weakness, however, is of temporary duration, as in the state of pregnancy, the teeth, as a rule, return to their normal character after the saliva soon loses its acidity and viscidity, the enamel and the dentine gradually regain their transparency and hardesss. The teeth become less sensitive at the necks, and the gums, usually independently of treatment, are restored to a healthy state. When, however, through chronic diseases of the kidneys, liver and nervous diseases, the health of the patient fails and you soon begin to notice a marked change in the teeth and their surroundings, sometimes before the patient is aware of his ailment.

Thus in making a diagnosis, you must carefully consider the individual characteristics of the patient, the normal standard of his or her health, and ascertain, if possible, whether the system is deranged by temporary or constistutional disturbances, such as menstruation, pregnancy, anemia, chronic diseases of the nervous system, of the liver, or of the kidneys, the effects of external or hereditary influence, etc.

After the diagnosis, the dentist should do his own prescribing and should undertand the therapeutic action of the different drugs and chemicals so as to know exactly their therapeutic effects and what chemical changes are liable to take place after their administration.

In taking up bacteriology, we have a subject of most vital importance to the dentists. You read endless amounts of literature about how to use the different antiseptics for the protection of bodies from putrefactive processes, but know comparatively little about their chemical composition, how they act, and the conditions under which they act, etc.

Bacteria, as you all know, are the active agents in decomposition, and that these bacteria come from without and are not developed spontaneously in the decomposing fluid or substance. Bacteria appear structureless. They consist of a peculiar form of protoplasm known as mycoprotein, the composition of which varies in different species. It is probable from their great resistance to alkalies and dilute acids, that bacteria possesses a cell membrane formed of some carbo-hydrate allied to cellulose.

During the formation of spores and after the action of tincture of iodine, which stains and causes shrinking of the protoplasm, a fine membrane may be actually seen. It is very elastic and seems to form the inner layer of a gelatinous envelope, by more or less of which all bacteria are surrounded.

They exist chiefly on nitrogenous compounds, which even when insoluble in water, are rendered fluid by the agency of bacteria. When the nitrogenous substances in which the bacteria multiply is completely used up, these bodies pass into a stationary condition and become congregated into gelatinous colonies in which they can resume their activity under favorable conditions.

All fluids containing decomposing organic substances have some of these bodies present, and usually in great numbers. When such fluids evaporate, the bacteria germs are carried into the air, and hence become causes of putrefaction in nitrogenous substances exposed to the air, and may serve as propagators of contagious diseases. Indeed, according to the modern doctrine of the production or generation of disease by germs, the contagious diseases, etc., are produced by certain specific organisms or poisons. In septicemia, for example, an absorption has occurred of certain fluids whose decomposition has been induced by bacteria, and the system is proportionately poisoned. Fluids also that have been subjected to the action of these bacteria, but subsequently freed from them, have been proved to act as septic poisons to the system. Accordingly, it would seem that the bacteria themselves are not the germs of disease, but rather possess the power, under suitable conditions, of generating the contagium.

By inoculating sheep with the prepared blood of cattle that have died of splenic fever, they were protected from taking that disease. This protection is undoubtedly due to the organism introduced by inoculation, exhausting the substance in the body which affords them nourishment, and that when the animals are attacked by the real disease, the disease germs, however, introduced, find no food and accordingly perish.

Many chemical substances are inimical not only to the growth but also to the very existance of organisms. It has been suggested that the term "antiseptic" should be reserved for those substances which prevent their growth, but which do not cause their destruction while those which actually kill the germs should be called "germicides." But the distinction is not an absolute one. Thus most germicides can be so diluted that they act only as antiseptics though the converse is not equally true.

Mercuric chloride is, on the whole, the most powerful germicide known. A solution of 1-1000 will kill any spores in half an hour. Its power is increased by the addition of five times as much salt or of hydrochloric acid, while it is seriously diminished by the presence of an albuminous fluid and absolutely destroyed by the addition of alkalies, and therefore soap.

A 1-20 watery solution of carbolic acid rapidly destroys fully-developed bacteria, but takes a few days to kill the more resistant spores. The addition of hydrochloric acid (half as much), increases its germicidal value. It will be readily understood that the germicidal power of any substance must, to some extent, depend on the nature of the organism, on the vitality of the particular specimen in question, on any physical conditions that may interfere with immediate contact, and on the presence of any neutralizing or incompatible substances.

